# External Hemin as an Inhibitor of Mitochondrial Large-Conductance Calcium-Activated Potassium Channel Activity

**DOI:** 10.3390/ijms232113391

**Published:** 2022-11-02

**Authors:** Agnieszka Walewska, Adam Szewczyk, Piotr Koprowski

**Affiliations:** Laboratory of Intracellular Ion Channels, Nencki Institute of Experimental Biology, Polish Academy of Sciences, 02-093 Warsaw, Poland

**Keywords:** BK_Ca_ channel, mitochondria, hemin, heme-binding site, hydrogen sulfide

## Abstract

The mitochondrial large-conductance calcium-activated potassium channel (mitoBK_Ca_) is located in the inner mitochondrial membrane and seems to play a crucial role in cytoprotection. The mitoBK_Ca_ channel is regulated by many modulators, including activators, such as calcium ions and inhibitors, such as heme and its oxidized form hemin. Heme/hemin binds to the heme-binding motif (CXXCH) located between two RCK domains present in the mitochondrial matrix. In the present study, we used the patch-clamp technique in the outside-out configuration to record the activity of mitoBK_Ca_ channels. This allowed for the application of channel modulators to the intermembrane-space side of the mitoBK_Ca_. We found that hemin applied in this configuration inhibits the activity of mitoBK_Ca_. In addition, we proved that the observed hemin effect is specific and it is not due to its interaction with the inner mitochondrial membrane. Our data suggest the existence of a new potential heme/hemin binding site in the structure of the mitoBK_Ca_ channel located on the mitochondrial intermembrane space side, which could constitute a new way for the regulation of mitoBK_Ca_ channel activity.

## 1. Introduction

Potassium channels are the most common ion channels found in the cell membranes of many organisms—from viruses, bacteria, and plants to humans [1,2,3,4,5]. They are found not only in the cell membrane but also in the membrane surrounding the cell nucleus, endoplasmic reticulum, lysosomes, Golgi apparatus, and inner mitochondrial membrane [6]. The large-conductance calcium-activated potassium (BK_Ca_) channel is found in most types of mammalian cells [7,8,9,10] and plays a key role in a variety of physiological processes. Briefly, BK_Ca_ channels play a role in the regulation of vascular blood pressure [11], bladder function [12], release of neurotransmitters [13,14], proper functioning of the circadian clock [15], and many other processes.

The BK_Ca_ channel is a protein consisting of four α subunits that form the channel pore with a conductivity from 200–300 pS [16]. Each α subunit contains seven transmembrane segments, a short N-terminus located on the extracellular side, and a large C-terminus with two regulatory domains of K^+^ conductance (RCK1 and RCK2) [17,18] located in the cytoplasm. BK_Ca_ channel activity is regulated by various stimuli, including voltage [19], membrane tension [20,21,22], and Ca^2+^ [23,24,25,26]. It was shown that two binding sites for Ca^2+^ are present in the structure of the BK_Ca_ channel, and both of them are located on the cytoplasmic part of the channel [27,28,29,30].

BK_Ca_ channel activity is regulated by several activators and inhibitors. The known endogenous activators of the BK_Ca_ channel, apart from Ca^2+^, include phosphatidylinositol 4,5-bisphosphate (PIP2) [31] and polyunsaturated fatty acids [32] or corticosterone [33]. Additionally, many synthetic activators have been developed, such as the potassium channel openers NS1619 [34], NS11021 [35], and CGS7184 [36]. Naturally occurring inhibitors of the BK_Ca_ channel include the indole alkaloid paxillin (Pax) [37], iberiotoxin (IbTx) [38], and charybdotoxin (ChbTx) [39]. The binding sites for IbTx and ChbTx in the BK_Ca_ channel are located extracellularly [40,41]. Additionally, heme and its oxidized form, hemin, inhibit the activity of the plasmalemmal BK_Ca_ channel by binding with the heme-binding motif (HBM) located in the linker between the RCK1 and RCK2 domains [42,43]. HBM is composed of two cysteines (C612, C615) and one histidine (H616) in the CXXCH motif [42,43]. The same motif is present in cytochrome c [44,45,46]; however, despite this, the mechanisms of heme-binding within the BK_Ca_ channel and cytochrome c are slightly different. Heme c is attached to cytochrome c by two thioether bonds formed between the heme vinyl groups and cysteine sulfur atoms. In addition, the iron ion of heme c is ligated by two amino acid side chains from the protein, where the proximal ligand is histidine from the HBM, and the distal ligand is provided elsewhere on the polypeptide [44,47]. However, heme b is only transiently bound in the BK_Ca_ channel, supposedly by iron ion coordination with the imidazole ring of histidine [42,48]. It was also shown that cysteine (C615) is important for the inhibition of BK_Ca_ channel activity by heme; however, details of this interaction are not known [42].

**Figure 1:**
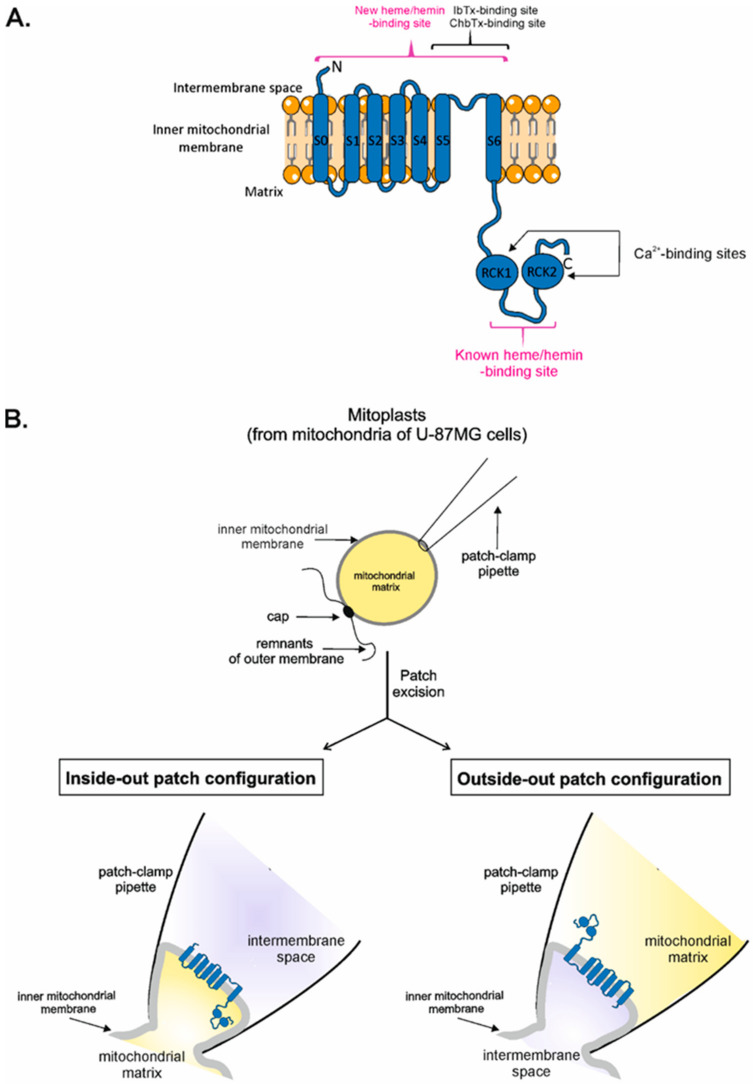
Topology of the mitoBK_Ca_ channel α subunit and scheme of patch-clamp experiments in different configurations. (**A**) Schematic topology of mitoBK_Ca_ α subunit with marked binding sites for selected modulators of the mitoBK_Ca_ channel activity. Binding sites for Ca^2+^ in the mitoBK_Ca_ channel are located on the mitochondrial matrix side, while iberiotoxin- and charybdotoxin-binding sites are present on the external, intermembrane space side of the channel. The known heme-binding site (CXXCH) is located in the linker between RCK domains in the C-terminus, while a new hypothetical external heme-binding site is located on the intermembrane space side of the mitoBK_Ca_ channel. (**B**) Schematic representation of patching of the mitoplast and patch-clamp experiments in the inside-out and outside-out modes. For details of patch-clamp measurements see the “*Materials and Methods*” section.

In addition to the cell membrane, the BK_Ca_ channel is also present in the inner mitochondrial membrane, i.e., mitoBK_Ca_ [49,50,51]. Several studies indicate that mitoBK_Ca_ channels play a central role in protecting the heart from ischemia. Pharmacological activation of these channels affects the generation of reactive oxygen species and mitochondrial Ca^2+^, thus, preventing cell death, likely by hindering the unchecked opening of the mitochondrial transition pore (reviewed in [52,53,54,55]). The mitoBK_Ca_ channel is formed by a DEC splice variant encoded by the *KCNMA1* gene [56]. MitoBK_Ca_ seems to play a crucial role in cytoprotection [57,58,59,60]. Patch-clamp experiments on mitoplasts indicate that intramitochondrial (matrix) Ca^2+^ regulates mitoBK_Ca_ activity; therefore, the C-terminus of the mitoBK_Ca_ channel with RCK domains faces the mitochondrial matrix, while the N-terminus is located in the intermembrane space [54] (Figure 1A). Similar to plasmalemmal BK_Ca_ channels, mitoBK_Ca_ is also inhibited by Pax [61,62,63], IbTx [50,64], ChbTx [57,65,66], and hemin [61,67], which proves the existence of an HBM in the C-terminus of the mitoBK_Ca_ channel (Figure 1A). However, there are no data regarding the regulation of the activity of the mitoBK_Ca_ channel by external (cytosolic) heme/hemin.

An important source of hemin in the human body is intracerebral hemorrhages, during which hemin penetrates the interstitial space and surrounds neurons, astrocytes, and other cells in the brain [68]. The inhibition of BK_Ca_ channels by hemin may be a potential mechanism for the neurotoxicity associated with intracerebral hemorrhages, e.g., by elimination of the cytoprotective properties of the mitoBK_Ca_ channel.

In this work, we applied hemin to the mitochondrial intermembrane space side and observed the inhibition of mitoBK_Ca_ channel activity. In addition, we investigated whether the mitoBK_Ca_ channel inhibited by hemin applied to the mitochondrial intermembrane space side is reactivated by NaHS, used as a hydrogen sulfide (H_2_S) donor, similar to results obtained for mitoBK_Ca_ channel regulation from the matrix side [69]. Our data suggest the existence of a new potential heme/hemin binding site in the structure of the mitoBK_Ca_ channel located on the mitochondrial intermembrane space side.

## 2. Results

### 2.1. Definitions

Inside-out configuration of the mitoBK_Ca_ channel—the mitochondrial matrix side of the channel is exposed to the recording chamber, where modulators of the channel activity are directly applied, while the mitochondrial intermembrane space side of the channel is facing the recording pipette (Figure 1B).

Outside-out configuration of the mitoBK_Ca_ channel—the mitochondrial intermembrane space side of the channel is exposed to the recording chamber, where modulators of the channel activity are directly applied, while the mitochondrial matrix side of the channel is facing the recording pipette (Figure 1B).

External hemin is applied from the mitochondrial intermembrane space side of the channel.

### 2.2. Vectorial Properties of mitoBK_Ca_ Channels

**Figure 2:**
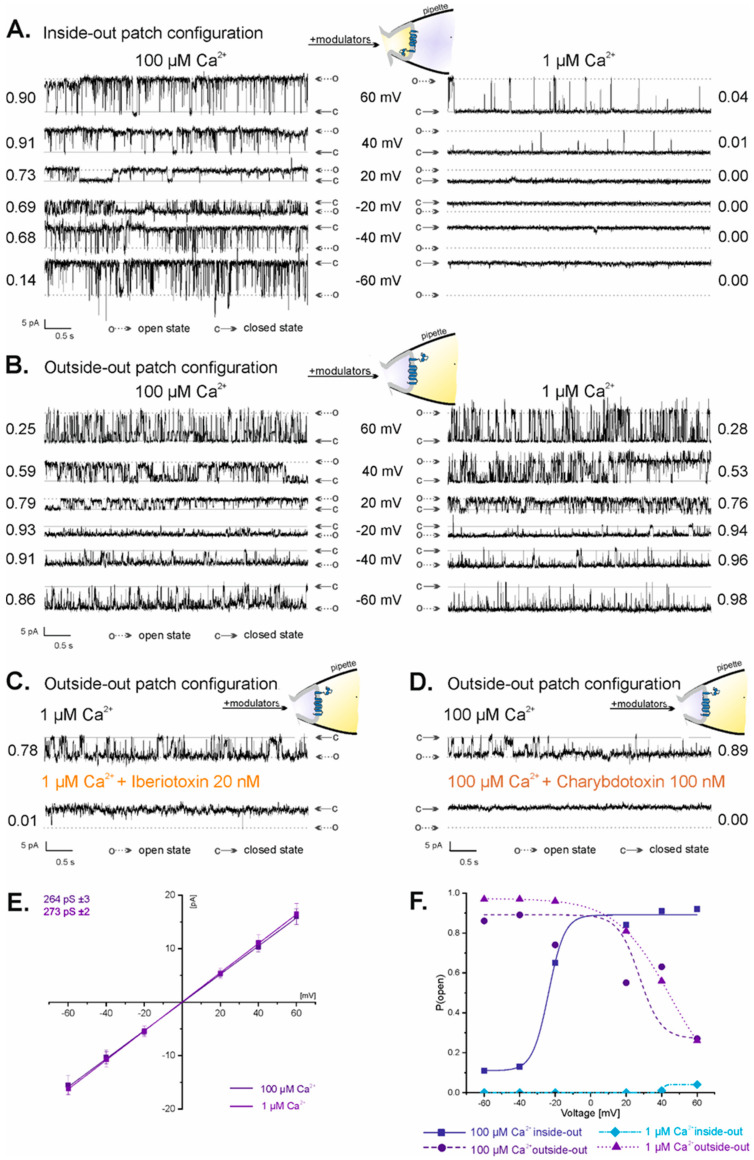
Properties of the mitoBK_Ca_ channel in the inside-out and outside-out configuration. (**A**) Regulation of the mitoBK_Ca_ channel in the inside-out configuration by batch Ca^2+^. Representative recordings of single-mitoBK_Ca_ channel activity at different voltages in high (100 µM Ca^2+^; left panel) and low (1 µM Ca^2+^; right panel) calcium solutions. (**B**) Lack of the regulation of the mitoBK_Ca_ channel in the outside-out configuration by bath Ca^2+^. Representative recordings of single-mitoBK_Ca_ channel activity at different voltages in high (100 µM Ca^2+^; left panel) and low (1 µM Ca^2+^; right panel) calcium solutions. (**C**,**D**) Inhibition of the activity of the mitoBK_Ca_ channel in the outside-out configuration by iberiotoxin and charybdotoxin. Channel activity was recorded at −40 mV. “c” denotes the closed state of the channel; “o” denotes the open state of the channel. (**E**) Current–voltage characteristics of the single-channel events in high and low calcium solutions. The conductance of the channel was equal to 264  ±  3 pS in 100 µM Ca^2+^ and 273 ± 2 pS in 1 µM Ca^2+^. (**F**) Analysis of the P(o) of the mitoBK_Ca_ channel at different voltages in inside-out and outside-out configurations in 100 µM Ca^2+^ and 1 µM Ca^2+^ solutions.

Two possible orientations of membrane patches derived from the inner mitochondrial membranes were observed in our patch-clamp experiments: inside-out and outside-out. They occurred randomly; however, the outside-out configuration was observed much less frequently (<20% of all experiments) than the inside-out configuration. Substances known to modulate mitoBK_Ca_ channel activity in a specific manner, for which localization of binding sites is defined, were used to distinguish between inside-out and outside-out mitoBK_Ca_ channel orientation. The mitoBK_Ca_ channels in the inside-out configuration were sensitive to calcium ions applied to the recording chamber—in 100 µM Ca^2+^, P(o) ranged from 0.14 at −60 mV to 0.90 at 60 mV (Figure 2A, left panel), while in 1 µM Ca^2+^, the channel was almost inactive at all voltages (P(o) = 0 at −60 mV to 40 mV and P(o) = 0.04 at 60 mV) (Figure 2A, right panel). In the outside-out configuration, where the Ca^2+^ binding sites located on the mitochondrial matrix side of the channel were inside the recording pipette, the mitoBK_Ca_ channel was not affected by changes in the concentration of calcium ions applied to the recording chamber (Figure 2B). In both 100 µM Ca^2+^ and 1 µM Ca^2+^, the activity of the mitoBK_Ca_ channel in the outside-out configuration was similar: P(o) in 100 µM Ca^2+^ ranged from 0.86 at −60 mV to 0.25 at 60 mV (Figure 2B, left panel), while in 1 µM Ca^2+^, it ranged between 0.98 and 0.28 at −60 mV and at 60 mV, respectively (Figure 2B, right panel). To additionally prove the outside-out configuration of the mitoBK_Ca_ channel, 20 nM IbTx (Figure 2C) and 100 nM ChbTx (Figure 2D), whose binding sites are located on the mitochondrial intermembrane space side of the channel, were applied. Both substances applied to the recording chamber completely inhibited the activity of the mitoBK_Ca_ channel (Figure 2C,D), proving that the intermembrane space side of the channel was exposed to the recording chamber. The conductance of the mitoBK_Ca_ channels in the outside-out configuration was similar to the previously reported conductance of the mitoBK_Ca_ channels in the inside-out configuration from the U-87MG cell line [70]. The channel conductance in the outside-out configuration was estimated to be 264 ± 3 pS in 100 µM Ca^2+^ (*n* = 10) and 273 ± 2 pS in 1 µM Ca^2+^ (*n* = 10) (Figure 2E). A comparison of the dependence of P(o) of the channel on voltages between inside-out and outside-out configurations in high and low Ca^2+^ concentrations is shown in Figure 2F. The dependence of P(o) of the mitoBK_Ca_ channel on voltages is opposite for inside-out (in 100 µM Ca^2+^: low values of P(o) from −60 to −40 mV, high values at voltages from −20 mV to 60 mV) and outside-out configurations (in both 100 µM Ca^2+^ and 1 µM Ca^2+^: high values of P(o) at voltages from −60 mV to 40 mV, low values at −60 mV) (Figure 2F). Altogether, the above results prove that the mitoBK_Ca_ channels observed in a fraction of the patch-clamp experiments were in the outside-out orientation.

### 2.3. Regulation of mitoBK_Ca_ Channels by External Hemin

**Figure 3:**
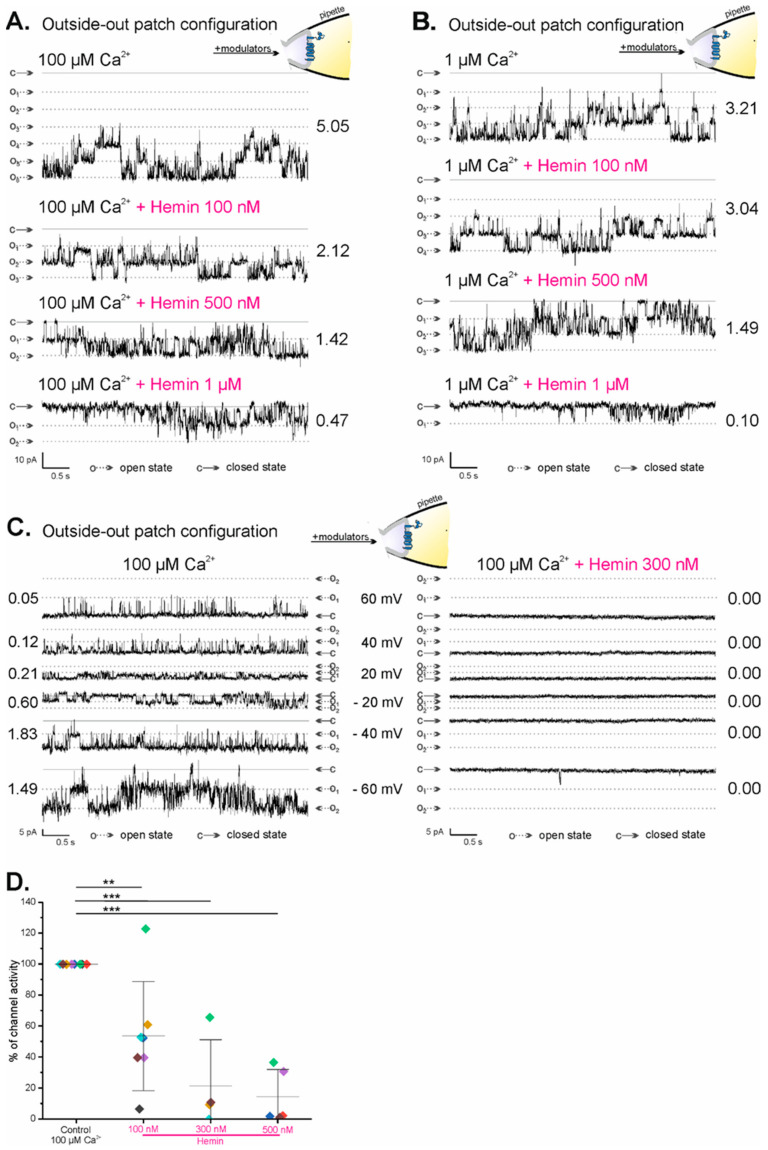
Regulation of the activity of the mitoBK_Ca_ channel in outside-out configuration by hemin. (**A**,**B**) Changes in the open probability of the mitoBK_Ca_ channel in multichannel patch during continuous experiments recorded at −40 mV. The gradual decrease in channel open probability after the application of increasing concentrations of hemin, both in 100 µM Ca^2+^ and 1 µM Ca^2+^. (**C**) Representative recordings of mitoBK_Ca_ channel activity in outside-out configuration at different voltages in the control conditions (100 µM Ca^2+^) and in the presence of 300 nM hemin; “c” denotes the closed state of the channel; “o_n_” denotes the open state of the channel with “*n*” indicating the number of open channels. (**D**) Statistical analysis of mitoBK_Ca_ channel activity in the outside-out configuration at −40 mV after the application of different hemin concentrations. The data are presented as the percentage of the channel activity with respect to the control conditions (mean ± SD with points representing a given repetition). A one-way ANOVA with Tukey means a comparisons test was used to identify any significant differences (*p* = 0.01–0.001 (**), *p* < 0.001 (***). The P(o) for the statistical analysis of channel activity was calculated for a one-minute-long recording starting two or more minutes after hemin application (unless stated otherwise).

Inhibition of the activity of the mitoBK_Ca_ channel by external hemin was observed in patch-clamp experiments in which mitoBK_Ca_ channels were active in the outside-out patch configuration. Figure 3A,B show representative recordings of mitoBK_Ca_ channels under control conditions—100 µM Ca^2+^ and 1 µM Ca^2+^, respectively—and after the addition of increasing hemin concentrations (100 nM, 500 nM, and 1 µM). NP(o) of the mitoBK_Ca_ channel in the outside-out configuration decreased from 5.05 (in 100 µM Ca^2+^) to 2.12 and 1.42 after the application of 100 nM and 500 nM hemin, respectively, to ultimately achieve a value of 0.47 in 1 µM hemin (Figure 3A). Similarly, NP(o) of the mitoBK_Ca_ channel decreased from 3.21 (in 1 µM Ca^2+^) to 3.04, 1.49, and 0.10 after the application of 100 nM, 500 nM, and 1 µM hemin, respectively, applied from the mitochondrial intermembrane space side of the channel (Figure 3B). In addition, it was shown that 300 nM hemin (Figure 3C, right panel) applied from the intermembrane space side on the mitoBK_Ca_ channel in control conditions (Figure 3C, left panel) inhibited its activity at all voltages, from −60 mV to 60 mV (Figure 3C, right panel). Figure 3D shows the statistical analysis of changes in the percent activity of the mitoBK_Ca_ channel in the outside-out configuration after the application of increasing hemin concentrations in comparison to control conditions. Dose-dependent inhibition of the mitoBK_Ca_ channel with 100 nM, 300 nM, and 500 nM hemin was observed, with corresponding mean percent channel activity values of 53.60% (±35.23, *n* = 7; *p* < 0.05 **), 21.47% (±29.84, *n* = 4; *p* < 0.05 ***), and 14.56% (±17.61, *n* = 5; *p* < 0.001 ***), respectively. The above results indicate that the mitoBK_Ca_ channel is inhibited by external hemin.

### 2.4. Regulation of mitoBK_Ca_ Channel Activity in the Outside-Out Configuration by NaHS and PPIX

**Figure 4:**
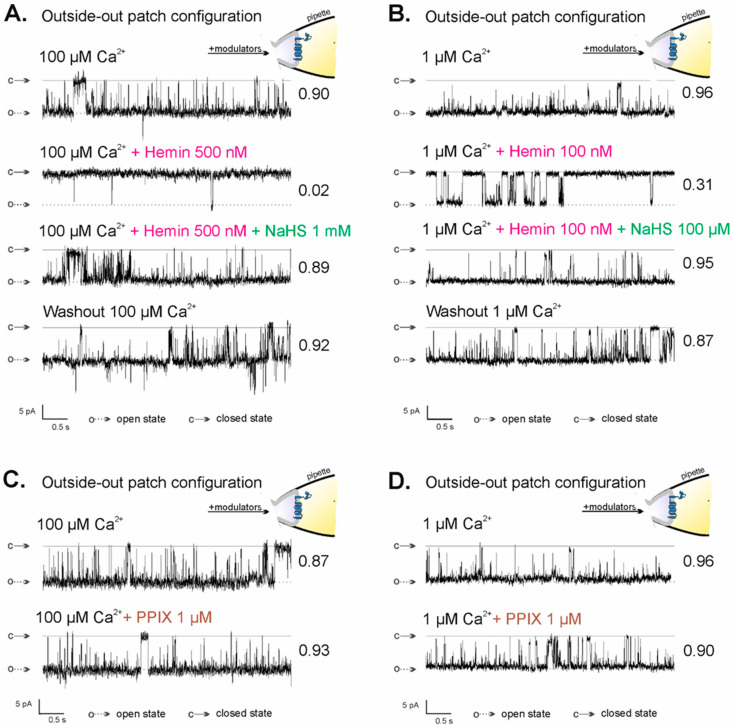
Regulation of the activity of the mitoBK_Ca_ channel in the outside-out configuration by NaHS and PPIX. (**A**,**B**) NaHS activates mitoBK_Ca_ channels in the outside-out configuration inhibited by external hemin. Representative recordings of single-mitoBK_Ca_ channel activity in outside-out configuration at −40 mV in control conditions ((**A**) 100 µM Ca^2+^ and (**B**) 1 µM Ca^2+^) and after the application of hemin followed by NaHS addition and washout step. (**C**,**D**) Lack of the effect of PPIX on the activity of the mitoBK_Ca_ channel in the outside-out configuration. Representative recordings of single-mitoBK_Ca_ channel activity in the outside-out configuration at −40 mV in control conditions ((**C**) 100 µM Ca^2+^ and (**D**) 1 µM Ca^2+^) and after the application of 1 µM PPIX; “c” denotes the closed state of the channel; “o” denotes the open state of the channel.

Previously, it was shown that hemin-inhibited mitoBK_Ca_ channels in the inside-out configuration were reactivated by NaHS, an H_2_S donor [69]. Because of the lack of a direct effect of H_2_S on the activity of the mitoBK_Ca_ channel and known H_2_S reactivity with iron ions, it was postulated that H_2_S binds to the iron cation of hemin [69]. To prove that the observed effect of hemin action on the activity of the mitoBK_Ca_ channel in the outside-out configuration is hemin-specific and results from the direct interaction of hemin’s iron ion with the mitoBK_Ca_ channel, analogous experiments were performed. NaHS activated hemin-inhibited mitoBK_Ca_ channels in the outside-out configuration. The effects of NaHS were similar at both low and high calcium ion concentrations: P(o) of the mitoBK_Ca_ channels increased from 0.02 (in 100 µM Ca^2+^ with 300 nM hemin) to P(o) = 0.89 after 1 mM NaHS administration (Figure 4A) and from 0.31 (in 1 µM Ca^2+^ with 100 nM hemin) to P(o) = 0.95 (Figure 4B). The activity of the mitoBK_Ca_ channel after the washout of both modulators remained at a high level: P(o) = 0.92 and P(o) = 0.87 after washout with 100 µM Ca^2+^ and 1 µM Ca^2+^, respectively. Porphyrins, including hemin, can bind lipid membranes, thereby changing their properties [71,72]. To exclude the effects of hemin due to its interaction with the membrane, experiments with protoporphyrin IX (PPIX) were performed (Figure 4C,D). PPIX is a precursor of heme/hemin that lacks a central iron ion, which is crucial in virtually all specific interactions of heme/hemin with proteins. Burton et al. showed that PPIX did not result in any change in the K_ATP_ currents, while heme increases whole-cell K_ATP_ currents. They concluded that the increases in current are specific to heme and are not a consequence of the porphyrin ring [73]. In the previous study, we have shown that PPIX applied in the inside-out configuration did not impact the activity of the mitoBK_Ca_ channel [69]. Here, we showed that 1 µM PPIX did not change the activity of the mitoBK_Ca_ channel, also in the outside-out configuration: P(o) in control conditions was 0.87 in 100 µM Ca^2+^ (Figure 4C) and 0.96 in 1 µM Ca^2+^ (Figure 4D), while after 1 µM PPIX administration, the values were 0.93 (Figure 4C) and 0.90 (Figure 4D), respectively. The above results indicate that the observed hemin effect is specific and it is not due to its interaction with the inner mitochondrial membrane, which suggests the existence of a new potential heme/hemin-binding site in the structure of the mitoBK_Ca_ channel located on the mitochondrial intermembrane space side.

## 3. Discussion

In general, the biophysical and pharmacological properties of mitochondrial potassium channels are considered to be similar to those present in the plasma membrane. However, localization in the inner mitochondrial membrane indicates an additional unique role of these channels. Numerous studies using activators and inhibitors indicate that the mitoBK_Ca_ channel participates in the cytoprotection of cardiac and neuronal cells [57,59,62,74,75,76]. For example, it was shown that an opener of BK_Ca_—NS1619—protected hearts against ischemic injury [57,59], and a natural flavonoid—quercetin, known as a mitoBK_Ca_ channel opener—also shows cardioprotective effects [77]. Interestingly, it is important to note that the activation of BK_Ca_ by specific K^+^ channel openers triggered the death of human glioma cells [78,79], suggesting the involvement of BK_Ca_ channels in cancer. Although direct involvement of mitoBK_Ca_ in cancer has not been shown, yet this is probable since the role of another mitochondrial channel—mitoKv1.3—in cancer is already established [80]. Together, the search for new activators, inhibitors, and mechanisms of the regulation of the activity of the BK_Ca_ channels might provide more possibilities for the development of therapeutic strategies. Despite that the properties of plasmalemmal and mitochondrial BK_Ca_ channels are very similar, differences in their regulation by hypoxia were observed previously [65]. In this study, we added to the list of differences in the modulation by hemin (see below).

Heme, a small organic molecule with a central iron ion, is partially synthesized in mitochondria [81] and plays a crucial role in numerous organismal processes [82,83]. Heme-containing proteins form a large and biologically important group, and they are found in all living species and carry out a wide variety of functions, for example, in oxygen transport (the globins), electron transfer (the cytochromes), and various heme-dependent catalytic processes (e.g., in the cytochrome P450s, nitric oxide synthases, peroxidases, and dioxygenases). In many heme proteins, e.g., soluble guanylyl cyclase or cytochrome c, heme is bound or coordinated in part by an amino acid sequence typically containing a histidine or cysteine residue, which acts as an axial fifth ligand (in addition to the four bonds provided by the nitrogen atoms of the protoporphyrin-IX ring to the iron center) to the redox-sensitive iron center, and water or a bound gas molecule acts as the sixth ligand [84]. Recent studies revealed a novel role of heme and its oxidized form, hemin, as modulators of potassium channel activity.

Except for the BK_Ca_ channel from the plasma membrane, which contains the conserved heme-binding amino-acid sequence motif CXXCH located between two RCK domains in the C-terminus [42], other potassium channels are also regulated by heme through its interaction with heme-binding motifs. One of them is the cardiac ATP-sensitive K^+^ channel (K_ATP_ channel), a hetero-octameric complex consisting of four pore-forming K^+^ channel subunits of the inward rectifier family (Kir6.2) and four regulatory sulfonylurea receptor subunits (SUR2A). It was shown that the cytoplasmic part of SUR2A contains the heme-binding motif CXXHX_16_H. Mutagenesis together with quantitative and spectroscopic analyses of heme-binding and single-channel experiments identified Cys628 and His648 as important for heme-binding [73]. Another potassium channel regulated by free intracellular heme is the Kv1.4 channel (voltage-gated K^+^), whose inactivation is mediated, among others, by the N-terminal protein structure [85,86]. Inspection of the Kv1.4 primary structure did not reveal classic heme-binding motifs, such as those found in cytochrome c (CXXCH). However, the N-terminal ball structure possesses a residue of His16 close to Cys13, forming a putative heme-responsive CXXH motif with an additional histidine residue at position 35. Sahoo et al. showed that in the Kv1.4 channel, heme is ligated by the side chain of C13 [87].

In all the above-mentioned channels, heme interacts with its cytoplasmic domains to modulate channel activity, and in each case, heme is suggested to bind a flexible region with a distinct motif. Although there are similarities in the modes of heme-binding across different channels, the functional consequences are not the same in each case, which is a clear indication of the potential versatility of heme-binding processes in ion channel control.

Until now, there were no data regarding heme-binding motifs in the extracellular parts of plasmalemmal potassium channels or in the intermembrane space side of the mitochondrial channels. In addition, Tang et al. indicated that hemin did not show any noticeable effect when applied to BK_Ca_ channels from the extracellular side [42]. Our studies showed, for the first time, that hemin inhibits the activity of the mitoBK_Ca_ channel when applied from the intermembrane space side. Differences in the above observations may result from the presence of different isoforms of the BK_Ca_ channel in the inner membrane of mitochondria and in the plasma membrane. There are known examples of different effects of the same modulator on BK_Ca_ and mitoBK_Ca_ channel activity. It was shown that H_2_S potentiated BK_Ca_ currents in human uterine artery smooth muscle cells [88]. H_2_S also increased the activity of BK_Ca_ channels in rat pituitary tumor cells [89]. In contrast, BK_Ca_ channels were inhibited by H_2_S in colonic smooth muscle cells [90] and human-induced pluripotent stem cell-derived mesenchymal stromal cells [91]. On the other hand, the activity of the mitoBK_Ca_ channel is not directly regulated by H_2_S [69]. Therefore, some differences in the isoforms, membrane composition, or local microenvironment may result in different sensitivities of BK_Ca_ and mitoBK_Ca_ channels to external hemin. However, the discovery of the differentiating factors requires further studies.

In summary, we provide single-channel functional data suggesting the presence of an additional new hemin-binding site in the mitoBK_Ca_ channel located in the mitochondrial intermembrane space. The binding of heme/hemin to this site may contribute to the overall response of mitochondria during the cytoprotection phenomenon.

## 4. Materials and Methods

### 4.1. Cell Culture

Astrocytoma U-87MG cells were cultured in DMEM (Laboratory of General Chemistry, Institute of Immunology and Experimental Therapy, Polish Academy of Sciences, Wroclaw, Poland) with 2 mM L-glutamine (Gibco, Carlsbad, CA, USA), 10% FBS (Gibco, Carlsbad, CA, USA), 100 U/mL penicillin (Sigma-Aldrich, St. Louis, MO, USA), and 100 μg/mL streptomycin (Sigma-Aldrich, St. Louis, MO, USA). Cells were cultured at 37 °C in a humidified atmosphere with 5% CO_2_. The cells were fed and reseeded, usually every fourth day.

### 4.2. Mitochondria Isolation

Mitochondria from the U-87MG cell line were isolated as previously described with modifications [69,92,93]. Briefly, the cells were washed with PBS (Laboratory of General Chemistry, Institute of Immunology and Experimental Therapy, Polish Academy of Sciences, Wroclaw, Poland), harvested by scraping, collected in PBS, and centrifuged at 800× *g* for 10 min. The cell pellet was resuspended and homogenized in isolation buffer (250 mM sucrose, 1 mM EGTA, 5 mM HEPES, pH = 7.2). The homogenate was transferred into an Eppendorf tube and centrifuged at 9200× *g* for 10 min. Next, the pellet was resuspended in isolation buffer and centrifuged at 780× *g* for 10 min. The supernatant containing mitochondria was moved to a new Eppendorf tube and centrifuged at 9200× *g* for 10 min. The mitochondrial pellet obtained after the last centrifugation was resuspended in a small volume of isolation buffer (20–100 µL). All manipulations were performed on ice, while all centrifugation steps were performed at 4 °C. Isolated mitochondria were kept on ice and used for patch-clamp experiments 4–6 h after isolation.

### 4.3. Mitoplast Preparation

Mitoplasts were prepared according to a previously described protocol [70]. Briefly, mitochondria (1–2 µL) isolated from the U-87MG cell line were incubated in 40 µL of hypotonic solution (5 mM HEPES, 100 μM CaCl_2_, pH = 7.2) for approximately 2 min to induce swelling and breakage of the outer membrane, followed by the addition of 10 µL of hypertonic solution (1.5 M sucrose, 30 mM HEPES, 100 μM CaCl_2_, pH = 7.2) to restore the isotonicity of the medium.

### 4.4. Patch-Clamp Experiments

Patch-clamp experiments were performed according to published protocols [70,93]. Briefly, the experiments were carried out in the inside-out or outside-out modes. In the inside-out mode, the matrix side of the channel was exposed to the recording chamber, while the intermembrane space side was facing the recording pipette. In the outside-out mode, the channel’s orientation was the opposite; the intermembrane space side of the channel was exposed to the recording chamber, while the matrix side of the channel was facing the recording pipette (Figure 1B). Freshly prepared mitoplasts (0.5–2 µL) were added by mixing directly within the recording chamber filled with a high Ca^2+^ solution (150 mM KCl, 10 mM HEPES, and 100 μM CaCl_2_ at pH = 7.2). Modulators of the channel activity were added directly to the recording chamber (in the inside-out and outside-out modes). The patch-clamp pipettes, made of borosilicate glass (Harvard Apparatus GC150–10, Holliston, MA, USA) with a resistance of ~15 MΩ, were filled with a high calcium solution. After the mitoplast caught and established the giga-ohm seal, the membrane patch was excised by tapping the pipette holder. The current was recorded using a patch-clamp amplifier (Axopatch 200B, Molecular Devices Corporation, San Jose, CA, USA), low-pass filtered at 1 kHz, and sampled at a frequency of 2.5 kHz. Clampfit 10.7 software in the single-channel search mode was used to calculate the probability of mitoBK_Ca_ channel opening (P(o)). Two types of patch-clamp recordings were performed: ten-second recordings at different voltages (from −60 mV to 60 mV, 20 mV intervals) and continuous recordings at −40 mV and 40 mV with different lengths depending on the type of experiment. Stock solutions of modulators of the activity of the mitoBK_Ca_ channel used in patch-clamp experiments were prepared in the concentrations mentioned below and then diluted to the desired concentrations. Hemin (Sigma-Aldrich, St. Louis, MO, USA) and protoporphyrin IX (PPIX, LifeTein LLC, Somerset, NJ, USA) were diluted in DMSO to a 10 mM concentration and stored at −20 °C. NaHS (Fluorochem Ltd., Hadfield, United Kingdom) was diluted in water to a 1 M concentration. NaHS stock solutions were prepared immediately before use. ChbTx (Alomone Labs, Jerusalem, Israel) and IbTx (Smartox Biotechnology, Saint Egrève, France) were diluted in water to 100 µM concentration and stored at −20 °C.

### 4.5. Statistical Analysis

One-way ANOVA with Tukey’s means a comparisons test was used to determine the statistical significance of the obtained results. A value of *p* < 0.05 was considered statistically significant. The following significance levels were adopted: 0.05–0.01 (*), 0.01–0.001 (**), and <0.001 (***).

## Data Availability

Not applicable.
